# Supplementary material for: MetaRibo-Seq measures translation in microbiomes
Source: Nat Commun. 2020 Jun 29;11:3268. doi: 10.1038/s41467-020-17081-z (PMC7324362; doi:10.1038/s41467-020-17081-z)
Supplement: Supplementary file 10 — Supplementary Data 7 [file 41467_2020_17081_MOESM10_ESM.zip › File2/Confidence_High_Taxonomy/63504_out.krona.html]

Javascript must be enabled to view this page.

members
magnitude
magnitudeUnassigned
count
unassigned
taxon
rank

63504\_out

4

2
4
superkingdom

phylum
1224
4

class
1236
4

order
91347
4

1
4

SRS019129\_contig\_number\_6305
543
family

3
561
genus

562
3

SRS017521\_contig\_number\_45170SRS146888\_contig\_number\_15329SRS147022\_contig\_number\_contig-100\_247.93781
species
